# Cyclophilin D Deficiency Rescues Axonal Mitochondrial Transport in Alzheimer’s Neurons

**DOI:** 10.1371/journal.pone.0054914

**Published:** 2013-01-31

**Authors:** Lan Guo, Heng Du, Shiqiang Yan, Xiaoping Wu, Guy M. McKhann, John Xi Chen, Shirley ShiDu Yan

**Affiliations:** 1 Department of Pharmacology & Toxicology and Higuchi Bioscience Center, School of Pharmacy, University of Kansas, Lawrence, Kansas, United States of America; 2 College of Chemistry and Chemical Engineering, Lanzhou University, Lanzhou, Gansu, People’s Republic of China; 3 Department of Neurosurgery, Physicians & Surgeons College of Columbia University, New York, New York, United States of America; 4 Department of Neurology, Memorial Sloan-Kettering Cancer Center, New York, New York, United States of America; Universidad de Sevilla, Spain

## Abstract

Normal axonal mitochondrial transport and function is essential for the maintenance of synaptic function. Abnormal mitochondrial motility and mitochondrial dysfunction within axons are critical for amyloid β (Aβ)-induced synaptic stress and the loss of synapses relevant to the pathogenesis of Alzheimer’s disease (AD). However, the mechanisms controlling axonal mitochondrial function and transport alterations in AD remain elusive. Here, we report an unexplored role of cyclophilin D (CypD)-dependent mitochondrial permeability transition pore (mPTP) in Aβ-impaired axonal mitochondrial trafficking. Depletion of CypD significantly protects axonal mitochondrial motility and dynamics from Aβ toxicity as shown by increased axonal mitochondrial density and distribution and improved bidirectional transport of axonal mitochondria. Notably, blockade of mPTP by genetic deletion of CypD suppresses Aβ-mediated activation of the p38 mitogen-activated protein kinase signaling pathway, reverses axonal mitochondrial abnormalities, improves synaptic function, and attenuates loss of synapse, suggesting a role of CypD-dependent signaling in Aβ-induced alterations in axonal mitochondrial trafficking. The potential mechanisms of the protective effects of lacking CypD on Aβ-induced abnormal mitochondrial transport in axon are increased axonal calcium buffer capability, diminished reactive oxygen species (ROS), and suppressing downstream signal transduction P38 activation. These findings provide new insights into CypD-dependent mitochondrial mPTP and signaling on mitochondrial trafficking in axons and synaptic degeneration in an environment enriched for Aβ.

## Introduction

Neurons are highly polarized cells with axons projecting from the cell body to transmit interneuronal information. Axons rely on axonal transport to deliver most essential proteins and membrane bound organelles [1], [2]. Among the many types of axonal transport cargo, mitochondria play an essential role in supporting synaptic activity and plasticity due to their ability to generate ATP and meticulously regulate local calcium homeostasis [3]–[5]. The saltatory and bidirectional transports of mitochondria accumulate axonal mitochondria around structures such as presynapses and growth cones where there are high energy demand and constant calcium fluctuation [6]–[8], suggesting the close relationship of mitochondrial function, transport and positioning [9].

Indeed, concomitant mitochondrial dysfunction and motility change has been observed in neurodegenerative diseases including Alzheimer’s disease (AD) [10]. As a major causative factor of AD, amyloid beta (Aβ) particularly its oligomeric form, exerts multiple effects on mitochondrial function including intra-mitochondrial Aβ accumulation, decreased mitochondrial respiration and membrane potential, impaired permeability transition, and increased production of mitochondrial reactive free radicals [11]–[19]. Our recent studies indicate that mitochondria at synapses including axonal mitochondria are early victims of Aβ toxicity along with alterations in axonal mitochondrial movement [20]–[22]. More recently, emerging studies accentuated alterations in axonal mitochondrial motility and dynamics in Aβ-rich environments and suggest axonal mitochondrial motility change is closely correlated to synaptic dysfunction in AD neurons [22]–[26]. It thus raises an intriguing question of whether Aβ-induced mitochondrial dysfunction contributes to changes in axonal mitochondrial motility. The specific mechanisms underlying Aβ-induced impairment in axonal mitochondrial transport have not been fully elucidated.

Cyclophilin D (CypD; gene: Ppif) is a key component of mitochondrial permeability transition pore (mPTP) that consists of the voltage dependent anion channel (VDAC) in the outer mitochondrial membrane, the adenine nucleotide translocase (ANT) in the inner membrane, and cyclophilin D (CypD) in the mitochondrial matrix. Release of CypD from matrix allows it to bind to the ANT and VDAC to trigger the opening of mPTP. The opening of mPTP constitutes non-selective, high conductance pore allowing transport of not only calcium by any solute below the pore size. This results in mitochondrial osmotic swelling and dissipation of mitochondrial membrane potential, reduced mitochondrial calcium retention capacity; decreased membrane potential; increased reactive oxygen species (ROS) production; and eventually, cell death [13], [27], [28]. Accordingly, we have demonstrated that the blockade of CypD significantly attenuates mPTP-related mitochondrial dysfunction and cognitive impairments in an AD mouse model [12], [29], suggesting the protective effect of CypD depletion against Aβ-associated synaptic degeneration. However, it remains unclear whether CypD-dependent mPTP leading to mitochondrial dysfunction is linked to Aβ-induced damage of axonal mitochondrial transport. If so, does blockade of mPTP via CypD depletion attenuate impaired mitochondrial transport and protect from Aβ toxicity? Given the close relationship of mitochondrial function with transport and the critical role of normal mitochondrial distribution in sustaining synaptic plasticity and strength, it is essential and logical to delineate the role of CypD in mitochondrial trafficking in axons in Aβ rich environment. The outcome of this study on axonal mitochondrial transport deepened our understanding of the impact of Cyclophilin D related perturbations on mitochondrial function and added to the body of CypD-dependent mechanisms underlying Aβ-induced mitochondrial and synaptic degeneration [12], [29].

The goal of the present study is to determine the effect of CypD on Aβ-induced axonal mitochondrial trafficking and synaptic damage. We demonstrate that the blockade of mPTP by CypD depletion rescues axonal mitochondrial trafficking and protects synapse from Aβ toxicity. The potential mechanisms underlying the protection of CypD deficiency on axonal mitochondrial trafficking are related to the suppression of Aβ-induced calcium perturbation and accumulation of axonal reactive oxygen species (ROS), and activation of downstream signal P38/MAPK pathway. These studies delineate new insights into the crosstalk of CypD-dependent mPTP and axonal mitochondrial transport, contributing to the synaptic pathophysiology in AD pathogenesis, especially related to Aβ-induced axonal mitochondrial injury.

## Methods

### Ethics Statement

This study was performed in strict accordance with the recommendations in the Guide for the Care and Use of Laboratory Animals of the National Institutes of Health. The protocol was approved by the Committee on the Ethics of Animal Experiments of the University of Kansas (IACUC protocol number: 203-01).

### Mice

Animal studies were approved by the Animal Care and Use Committee of University of Kansas in accordance with the National Institutes of Health guidelines for animal care. CypD homozygous null mice (*Ppif ^−/−^*) were kind gifts from Dr. Jeffery D. Molkentin. These animals were backcrossed 10 times into the C57BL6 background.

### Neuronal Culture

Mouse hippocampal neurons were cultured as previously described [20].

### Preparation of Oligomeric Aβ

Oligomeric Aβ1-42 was prepared as previously described [20].

### Axonal Mitochondrial Trafficking Recording and Data Analysis

These recordings were performed using previously reported protocols [20]. Axonal processes were determined by morphological characteristics and confirmed by Tau-1 retrospect staining as previously described [20]. To be more specific, a process that is two to three times longer than other processes stemming from the soma is considered to be an axon; besides, neurons were subjected to retrospect staining of Tau-1, which is abundant in axons and is widely accepted as axonal marker [20], [30]–[32]. The images were taken before and after treatment with 200 nM oligomer Aβ (24 hr), and/or 1 µM SB203580 (24 hr), 5 µM Probucol (24 hr) or 5 µM A23187 (30 minutes).

### Treatment of Cyclosporine A

Cyclosporin A (CsA, Sigma) at a final concentration of 500 nM was added to the cells 30 min prior to oligomeric Aβ treatment.

### Measurement of Mitochondrial Intra-axonal Ca^2+^ and ROS

Neurons were loaded with 1 µM Fluo-4 AM (Invitrogen) for 30 minutes to monitor changes in intracellular Ca^2+^ or 10 µM dichlorodihydrofluorescein (H2-DCF) to detect ROS. Fluorescence images were captured using the inverted Zeiss Axiovert 200 microscope with a stage based chamber (5% CO_2_, 37°C). Images were analyzed using Image J software. Background fluorescence was calculated by sampling the areas that were around the measured axons, but had no axons in these fields and background intensity was subtracted from the raw data.

### Immunoblotting Analysis

Samples were lysed in extraction buffer (10 mM Tris-HCl pH 7.4, 100 mM sodium chloride, 1 mM EDTA, 1 mM EGTA, 1 mM sodium fluoride, 20 mM sodium pyrophosphate, 2 mM sodium orthovanadate, 1%Triton X-100, 10% glycerol, 0.1% SDS, 0.5% deoxycholate, 1 mM PMSF) containing protease inhibitor cocktail (Calbiochem, set V, EDTA free), separated by SDS-PAGE (12% Bis-tris gel, Invitrogen), and then transferred to nitrocellulose membrane (Amersham). After blocking in TBST buffer (20 mM Tris-HCl, 150 mM sodium chloride, 0.1% Tween-20) containing 5% nonfat dry milk (Santa Cruz) for 1 hr at room temperature, the membrane was incubated and gently shaken overnight (at 4°C) with primary antibodies. This was followed by incubation with corresponding secondary antibody for 1 hr at room temperature. Chemiluminescence was detected using an electrochemiluminescence instrument (GE). The following antibodies were used in this experiment: mouse anti-phospho (pT180/pY182) -p38 (BD Biosciences), rabbit anti-p38 (Cell signaling technology), goat anti mouse IgG HRP conjugated and goat anti rabbit IgG HRP conjugated (Invitrogen). NIH image J software was utilized to analyze the scanned blots and to quantify the intensity of immunoreactive bands.

### Electrophysiological Recording

Recordings were performed at 30°C as described in the previous reports [33], [34]. Cells were continuously perfused with oxygen saturated artificial cerebrospinal fluid (ACSF) containing 1 µM TTX and 50 µM picrotoxin at a rate of 2 ml/min. Patch pipettes were filled with intrapipette solution containing 130 mM K-gluconate, 5 mM KCl, 10 mM HEPES, 2.5 mM MgCl_2_, 10 mM K-phosphocreatine, 4 mM MgATP and 0.6 mM EGTA, pH 7.3. Recording pipettes were prepared on a pipette puller (Sutter) and had a resistance of 2.5–4 MΩ when filled with intrapipette solution. Seal was performed on clearly visualized neuron bodies with 10–20 µm diameters. The spontaneous miniature excitatory postsynaptic currents (mEPSCs) were recorded at holding potential at −70 mV using MultiClamp 700A (Axon Instruments) and events were analyzed using Axon clampfit (Axon Instrument, version 8.2.0.235) and MiniAnalysis 6.0 (Synaptosoft).

### Neuronal Synaptic Density

Synaptic density of cultured neurons was measured by counting synaptophysin clusters attaching to neuronal dendrites and presented as the numbers of synaptophysin clusters per micron of dendrite. Neurons were fixed in 4% paraformaldehyde for 20 minutes and then blocked in 10% goat serum for 30 minutes. Synaptophysin was visualized by rabbit anti-synaptophysin IgG (Dako) followed by goat anti-rabbit IgG conjugated with TRITC (Sigma – Aldrich Corp.). Neuronal dendrites were visualized by mouse anti-MAP2 IgG (Boehringer Mannheim) followed by goat anti-mouse IgG conjugated with FITC (Sigma – Aldrich Corp.). Images were taken under a Biorad confocal and analyzed by NIH Image J program.

### Statistical Analysis

One-way ANOVA was used for repeated measure analysis. P<0.05 was considered significant. Post-hoc ANOVA was used when appropriate. STATVIEW statistics computer software was utilized. All data were expressed as mean ± Standard Error of the Mean (SEM).

## Results

### Loss of CypD Attenuates Aβ-induced Changes in Axonal Mitochondrial Motility and Dynamics

**Figure 1:**
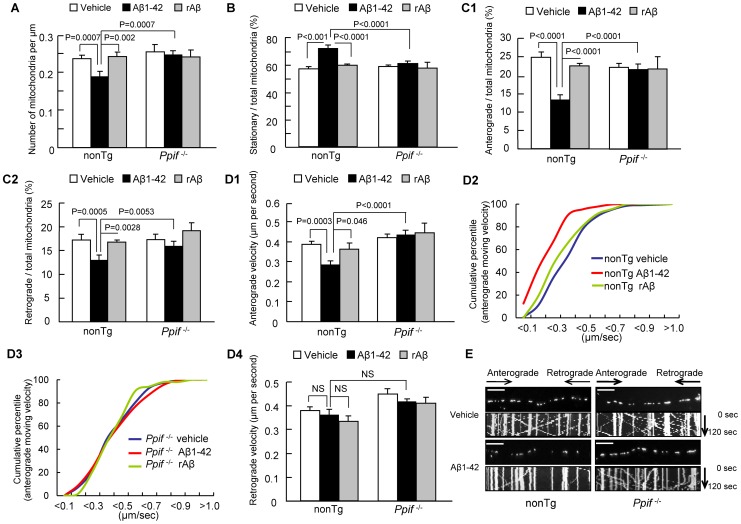
Loss of CypD protects axonal mitochondrial motility and dynamics from Aβ toxicity. (**A**) CypD depletion increased axonal mitochondrial density (numbers per micron of axon) in Aβ-treated neurons. rAβ: reversed Aβ42-1. There is no significant difference in the axonal mitochondrial density between vehicle-treated nonTg and *Ppif* ^−/−^ neurons. Data were collected from 3 independent experiments. (**B**) CypD depletion decreased the percentage of stationary mitochondria in Aβ-treated neurons. There were no significant changes in the percentage of stationary mitochondria between vehicle-treated nonTg and *Ppif* ^−/−^ neurons. Data were collected from 1380, 1074, 1410 mitochondria from vehicle, Aβ and rAβ groups in nonTg neurons, and 1634, 1505, 642 mitochondria in *Ppif* ^−/−^neurons, respectively, in 4 independent experiments. (**C**) CypD depletion restored the decrease in the percentage of anterograde mitochondria (**C1**) and retrograde mitochondria (**C2**) in Aβ-treated neurons. Data were collected from 4 independent experiments. (**D**) CypD depletion increased the velocity of mitochondrial movement. (**D1**) Aβ treatment deceased the velocity of anterograde movement of nonTg mitochondria but not in CypD-deficient (*Ppif* ^−/−^) mitochondria. Data were collected from 209, 141, 46 mitochondria from vehicle, Aβ and rAβ groups in nonTg neurons, and 158, 209, 52 mitochondria in *Ppif* ^−/−^neurons. (**D2–3**) The cumulative distribution data showed a left shift of the velocity of anterograde mitochondria when comparing the curve for Aβ-treated nonTg mitochondrial to *Ppif* ^−/−^ mitochondria. Data were collected from 3 independent experiments, respectively. (**D4**) Aβ treatment had no effect on the velocity of the retrograde mitochondria from both nonTg and *Ppif* ^−/−^ mice. (**E**) CypD depletion rescued axonal mitochondrial mobility. Images in the top portion of the panel and kymographs in the lower panel were generated from the live imaging movies. In the kymographs, the X axis represents the mitochondrial position and the Y axis is time. Vertical white lines represent stationary mitochondria and diagonal lines represent moving mitochondria. Anterograde movements are from left to right, retrograde movements are reversed. Scale bars represent 10 µm.

Axonal mitochondria are distributed along axons (Fig. S1) and decreased axonal mitochondrial density is a manifestation of disrupted mitochondrial trafficking. To determine the direct effect of CypD, we compared axonal mitochondrial distribution between cultured nonTg and CypD-deficient (*Ppif* ^−/−^) hippocampal neurons after exposure to 200 nM oligomeric Aβ1-42 or rAβ (reversed sequence of Aβ1-42) for 24 hours to mimic low *in vivo* levels and chronic Aβ insults in AD brain. Following Aβ treatment, nonTg neurons revealed significantly decreased axonal mitochondrial density (vehicle: 0.236±0.01/µm vs. Aβ: 0.188±0.01/µm) (**Fig. 1A**). In contrast, CypD depletion protected axonal mitochondrial density from Aβ toxicity (**Fig. 1A**; Aβ: 0.246±0.01/µm vs. vehicle: 0.254±0.019/µm). Axonal mitochondrial density showed no significant changes in vehicle-treated nonTg neurons when compared to *Ppif* ^−/−^ neurons (**Fig. 1A**), suggesting no effect of CypD depletion on axonal mitochondrial distribution without Aβ insults. The addition of control reversed Aβ42-1 (rAβ) did not affect axonal mitochondrial density in nonTg or *Ppif* ^−/−^ neurons (**Fig.** **1A**). These results indicate that CypD depletion preserves the organization of axonal mitochondrial distribution following Aβ insults.

We next investigated patterns of axonal mitochondrial movement. Mitochondria in the middle region of the axon were utilized for the study of movement patterns (movable or stationary) and movement direction (anterograde or retrograde) as previously described [20]. Mitochondria with displacement more than its length (∼2 µm) during a 120 second recording were considered to be movable; less movement was considered as ‘stationary.’ Among the mitochondria that showed movement, those exhibiting displacement towards the distal end of the axon at the end of the recording period were termed anterograde mitochondria, while those showing movement to the proximal end were termed retrograde mitochondria.

To objectively examine movement changes following Aβ treatment, we first measured baseline (vehicle treatment) movement patterns. The percentage of stationary mitochondria among total mitochondria in nonTg neurons was comparable to those in *Ppif* ^−/−^ neurons (**Fig.** **1B** **;** nonTg: 58.30±1.32% vs. *Ppif* ^−/−^: 60.22±0.92%), suggesting no effect of CypD depletion on normal docking of mitochondria. However, the percentage of stationary mitochondria increased by 1.3 fold in Aβ–treated nonTg neurons (**Fig. 1B, 1E** **;** Aβ: 73.40±2.34% vs. vehicle: 58.30±1.32%), but not in *Ppif* ^−/−^ neurons (**Fig. 1B, 1E** **;** Aβ: 62.30±1.45% vs. vehicle: 60.22±0.92%). These data indicate that the absence of CypD reverses Aβ-induced impairments in mitochondrial trafficking within axonal processes.

We then analyzed the direction of mitochondrial transport. Consistent with previous results [20], [24], [26], Aβ treatment significantly reduced the percentage of anterograde (**Fig. 1C** **1, 1E;** from 24.8±1.44% to 13.1±1.34%) and retrograde mitochondria (**Fig. 1C** **2, 1E;** from 17.52±1.28% to 12.91±0.92%) compared to vehicle-treated nonTg or *Ppif* ^−/−^ neurons. CypD-deficient neurons showed increases in both anterograde and retrograde mitochondrial movement in the face of Aβ toxicity as compared to Aβ-treated nonTg neurons (**Fig. 1C** **1–2, 1E;** anterograde: 21.55±1.59%; retrograde: 16.15±0.87% ).

Next, we examined the velocity of mitochondrial movement. Compared to vehicle-treated control, Aβ treatment decreased anterograde velocity of nonTg mitochondria by 26% (**Fig. 1D** **1, 1E;** Aβ: 0.287±0.018 vs. vehicle: 0.388±0.016 µm/sec), while the anterograde velocity of *Ppif* ^−/−^ mitochondria was preserved in conditions of Aβ toxicity (**Fig. 1D** **1, 1E;** 0.435±0.022 µm/sec). Vehicle treatment alone for nonTg and *Ppif* ^−/−^ mitochondria demonstrated comparable anterograde velocity (**Fig. 1D** **1;** nonTg: 0.388±0.016 vs. *Ppif* ^−/−^0.422±0.017 µm/sec). Analysis of cumulative distribution data revealed a leftward shift in the velocity curve for Aβ-treated anterograde nonTg mitochondria (**Fig. 1D** **2**), while velocity in Aβ-treated *Ppif* ^−/−^ anterograde mitochondria was not shifted as compared with vehicle-treated mitochondria (**Fig. 1D** **3**). Consistent with our previous results [20], Aβ treatment did not significantly impact the velocity of nonTg retrograde mitochondria when compared to vehicle-treatment (**Fig. 1D** **4**; vehicle: 0.381±0.016 vs Aβ: 0.362±0.032 µm/sec). Further, the velocity of *Ppif* ^−/−^ retrograde mitochondria was also not affected by Aβ insults (**Fig. 1D** **4**; vehicle: 0.450±0.022 vs Aβ: 0.419±0.015 µm/sec). As a control, the addition of rAβ did not significantly change directional mitochondrial movement in nonTg or *Ppif* ^−/−^ neurons (**Fig. 1B–1D** **4**). Taken together, these data indicate that CypD depletion significantly protects directional mitochondrial transport from the effects of Aβ toxicity.

### Effect of CypD Depletion on Aβ-instigated Axonal Mitochondrial Fragmentation

**Figure 2:**
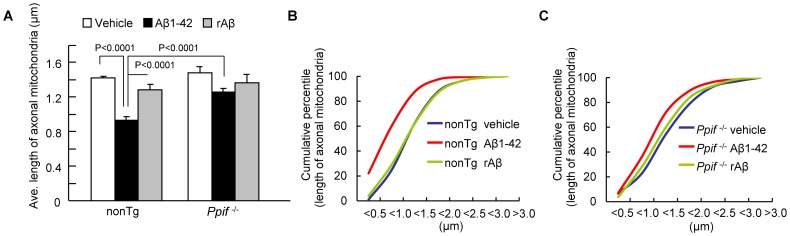
Effect of CypD on Aβ-induced changes in axonal mitochondrial morphology. (**A**) The average length of axonal mitochondria decreased in Aβ-treated nonTg neurons, but was largely preserved in *Ppif* ^−/−^ neurons. Data were collected from 3 independent experiments. (**B, C**) Cumulative distribution data showed that Aβ treatment caused a remarkable increase in fragmentation of small mitochondria and a decrease in the numbers of long mitochondria in nonTg neurons; this was partially attenuated in *Ppif* ^−/−^neurons.

To evaluate changes in mitochondrial morphology, we measured the average length of axonal mitochondria. Aβ treatment decreased the average length of nonTg axonal mitochondria by 34.3% (**Fig. 2A** **;** 1.421±0.022 µm in vehicle groups vs. 0.933±0.037 µm in Aβ-treated groups). Cumulative distribution data showed that Aβ treatment caused a remarkable increase in the number of small mitochondria and a decrease in the number of long mitochondria in nonTg neurons (**Fig. 2B**). Although *Ppif* ^−/−^ mitochondria demonstrated a 14.9% decrease in average length following Aβ exposure as compared to the corresponding in vehicle groups (**Fig. 2A** vehicle: 1.483±0.071 vs. Aβ: 1.256±0.043 µm), the average length of Aβ superimposed *Ppif* ^−/−^ mitochondria was better preserved than that of Aβ-treated nonTg mitochondria (**Fig. 2A–C** **;** *Ppif* ^−/−^:1.256±0.043 µm vs. nonTg: 0.933±0.037 µm). No significant difference was found in an average length when comparing vehicle-treated nonTg to *Ppif* ^−/−^ axonal mitochondria (**Fig. 2A**). The rAβ as a control did not alter axonal mitochondrial length (**Fig. 2A–C**). These results suggest that Aβ toxicity leads to increased axonal mitochondrial fragmentation and importantly, this effect is significantly attenuated by CypD depletion.

### CypD-associated Axonal Calcium Perturbation Alters Axonal Mitochondrial Transport

**Figure 3:**
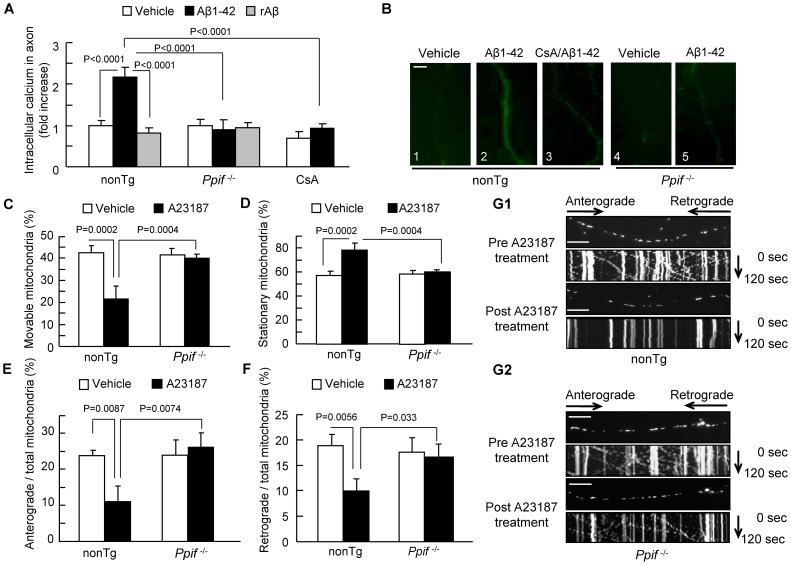
Effect of CypD depletion on Aβ-induced intra-axonal calcium elevation. (**A**) Aβ-treated nonTg hippocampal neurons displayed an increase in axonal calcium levels. CypD-deficient or CsA-treated (500 nM for 24 hours) neurons diminished elevated levels of calcium. rAβ had no effect on axonal calcium levels. Data were derived from 3 independent experiments. (**B**) Representative images of axonal calcium staining in nonTg and *Ppif* ^−/−^hippocampal neurons at indicated treatment. Scale bar represents 2 µm. (**C–G2**) Effect of CypD depletion on calcium ionophore (A23187)-impaired axonal mitochondrial motility. NonTg and *Ppif* ^−/−^hippocampal neurons were exposed to A23187 (5 µM for 30 min) and subjected to recording of axonal mitochondrial movements including movable (**C**), stationary (**D**), anterograde (**E**) and retrograde (**F**) mitochondria. *P<0.05 vs. other groups of neurons. (**G1–G2**) The kymograph of axonal mitochondrial movement in nonTg (**G1**) and *Ppif* ^−/−^ (**G2**) neurons before and after A23187 treatment. A23187 treatment resulted in less movement than the vehicle-treated group. *Ppif* ^−/−^ neurons revealed increased moving traces compared to nonTg neurons in the presence of A23187. Scale bar represents 10 µm.

Aβ has been shown to instigate intra-neuronal calcium elevation [35], [36], and the elevated intra-neuronal calcium is known to inhibit mitochondrial transport [37], [38]. Given that blockade of CypD-mediated mPTP formation significantly increases mitochondrial calcium buffering capability to maintain intra-cellular calcium homeostasis [12], [27], we evaluated whether CypD deficiency protects axonal mitochondrial motility by stabilizing intra-neuronal calcium in Aβ-insulted neurons. First, we measured intra-axonal calcium levels in Aβ-treated neuron as compared with vehicle-treated neurons by quantifying the staining intensity of Fluo-4, a cytoplasmic calcium indicator, and then evaluated the effect of CypD blockade. As shown in **figure 3**, Aβ-treated nonTg axons had a 2.3-fold higher Fluo-4 intensity than vehicle-treated nonTg axons (**Fig. 3A–B** **1–2**), while axons treated with cyclosporine A (CsA), a pharmacological inhibitor of CypD, (**Fig. 3A–B** **3**) or genetic CypD-deficient axons (**Fig. 3A–B** **4**-**5**) showed no significant change in calcium levels in the presence of Aβ. These results demonstrate that blockade of CypD attenuates Aβ-induced intra-neuronal calcium elevation and maintains intra-neuronal calcium homeostasis.

To further validate the role of CypD blockage on Aβ-induced calcium elevation as shown in Fig. 3A–B on abnormal axonal mitochondrial transport, we evaluated the direct effect of calcium overload on mitochondrial transport. Because CypD deficiency protects cell death from A23187-induced Ca^2+^ overload, a strong inducer of calcium elevation in intact cells [27], we assessed the effect of CypD depletion on A23187-mediated alterations in axonal mitochondrial transport. Neurons were exposed to 5 µM Calcium Ionophore (A23187). Axonal mitochondrial transport was recorded pre- and post-treatment with A23187 in the same neurons. Thirty minutes after A23187 treatment, the total of movable nonTg axonal mitochondria decreased by ∼50% (**Fig. 3C, 3G** **1**; 20.02±5.12% in post-treatment vs. 42.54±3.31% in pre-treatment). As a result, the percentage of stationary mitochondria was significantly increased (**Fig. 3D, 3G** **1;** 57.46±3.31% in pre-treatment vs. 79.98±5.12% in post-treatment). Similarly, A23187 treatment reduced anterograde and retrograde movement of mitochondria by ∼54% (**Fig. 3E, 3G** **1;** 23.73±1.56% in pre-treatment vs. 10.79±4.02% in post-treatment) and ∼50% (**Fig. 3F, 3G** **1;** 18.81±2.18% in pre-treatment vs. 9.23±2.39% in post-treatment), respectively. Notably, *Ppif* ^−/−^ axonal mitochondria are resistant to A23187-altered mitochondrial movement when evaluated for the percentage of movable vs. stationary mitochondria, anterograde or retrograde movement **(** **Fig. 3C–G** **)**. These results indicate that calcium imbalance plays a role in axonal mitochondrial trafficking and that CypD depletion protects against calcium-mediated disruption of axonal mitochondrial transport and motility.

### Effect of CypD Deficiency on ROS-instigated Alterations in Axonal Mitochondrial Transport

**Figure 4:**
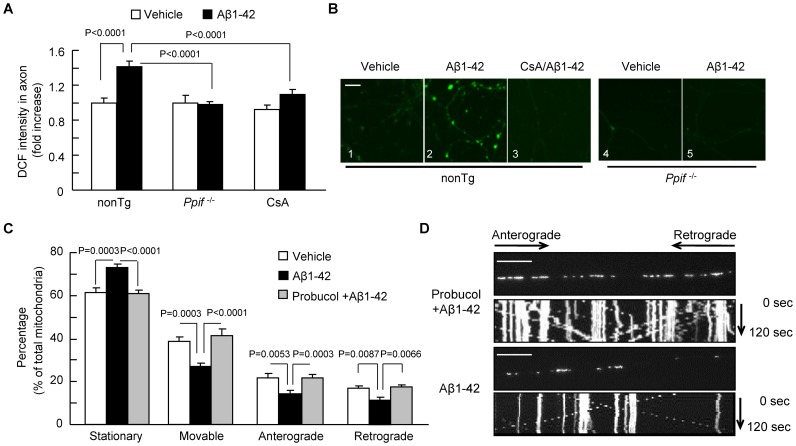
CypD depletion attenuates Aβ-induced intra-axonal ROS elevation. (**A**) Quantification of DCF intensity in nonTg- or *Ppif* ^−/−^ hippocampal neurons treated with vehicle or Aβ. Addition of CsA (500 nM) to cells for 24 hours reduced the DCF intensity. Data were derived from 3 independent experiments. (**B**) Representative images of axonal DCF staining in nonTg and *Ppif* ^−/−^ hippocampal neurons for the indicated treatment. Scale bar is 10 µm. (**C–D**) Effect of antioxidant (Probucol) on Aβ-induced axonal mitochondrial motility. (**C**) Administration of Probucol (5 µM, 24 hours) ameliorated changes in Aβ-induced axonal mitochondrial motility. (**D**) Kymograph images show the protected effects of axonal mitochondrial moving traces following Probucol treatment. Scale bar is 10 µm.

Because mitochondria are a major site of reactive oxygen species (ROS) production and because the formation of CypD-mediated mPTP triggers mitochondrial ROS generation, we next examined whether increased ROS generation contributes to impaired axonal mitochondrial transport. We first measured intra-axonal ROS levels using 2′7′-dichlorofluorescein diacetate (DCF-DA) fluorescent probe as an indicator of intracellular ROS in Aβ-treated axons for the comparison with vehicle-treated axons. Aβ-treated nonTg neurons revealed significantly higher DCF-DA intensity than the vehicle-treated group (**Fig. 4A, 4B** **1–2**), while Cyp D blockade produced by the addition of CsA (**Fig. 4A, 4B** **3**) or genetic deletion of CypD (**Fig. 4A, 4B** **4–5**) significantly blunted Aβ-induced increase in axonal DCF-DA intensity. These results indicate that blockade of CypD attenuates axonal ROS production or accumulation following Aβ insults.

To further evaluate whether increased axonal ROS production contributes to Aβ- mediated alterations observed in axonal mitochondrial trafficking, we examined the effects of the antioxidant Probucol on Aβ-impaired mitochondrial movement. Treatment with Probucol completely rescued the reduced percentage of movable mitochondria following Aβ treatment (**Fig. 4C–D** **;** from 26.87±1.59% to 41.53±2.86%). Accordingly, probucol treatment protected against Aβ-induced disruption of anterograde (from 14.44±1.40% to 21.83±1.28%) and retrograde mitochondrial movement (from 12.23±1.61% to 17.36±1.23%) (**Fig. 4C–D**).

### CypD-dependent Activation of p38 MAP Kinase Underlies the Axonal Mitochondrial Injury

**Figure 5:**
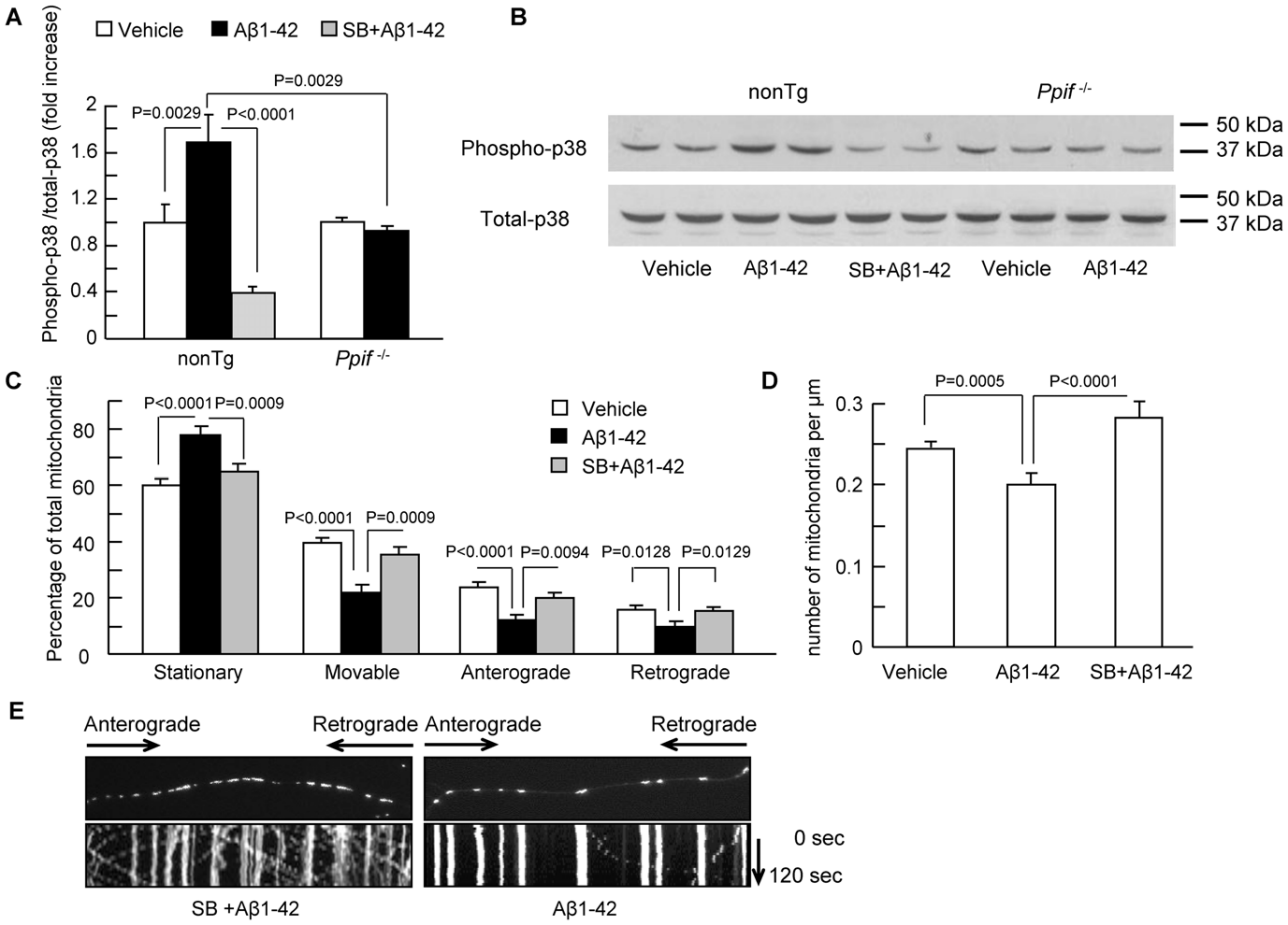
Effect of CypD on Aβ-induced activation of p38 MAP kinase and axonal mitochondrial motility. (**A**) Quantification of phospho-p38 immunoreactive bands (pT180/pY182) in hippocampal neurons treated with vehicle, Aβ, or SB203580 (SB, 1 µM) plus Aβ, respectively, which was normalized for the total p38. (**B**) Representative immunoblots for phospho- and total-p38. (**C–E**) Administration of p38 inhibitor, SB203580 (1 µM, 24 hours) to cells ameliorated Aβ-induced axonal mitochondrial motility changes (**C**) and mitochondrial density (**D**). (**E**) Kymographs showed the protected effects of axonal mitochondrial movement after SB203580 treatment. Scale bar is 10 µm.

It is known that Aβ activates a variety of kinases including p38/MAP kinase [39], [40] and that impaired mPTP leads to activation of p38 [41]. ROS and calcium are inducers for activation of p38/MAP kinase [42]–[44]. P38 activation inhibits fast axonal transport (FAT) by phosphorylation of kinesin (motor protein associated with FAT), which are respectively responsible for axonal mitochondrial transport [45], [46]. We therefore examined the relatively unexplored role of CypD-dependent mPTP in activation of p38 on Aβ-mediated axonal mitochondrial damage. To do so, we first analyzed the effect of CypD on Aβ-induced phosphorylation of p38 by immunoblotting. As shown in **figure 5A–B**, Aβ-treated nonTg neurons exhibited significantly increased levels of p38 phosphorylation compared to vehicle-treated nonTg neurons **(** **Fig. 5A** **)**. Addition of a specific p38 inhibitor (SB203580) to neurons completely suppressed p38 phosphorylation **(** **Fig. 5A** **)**. Interestingly, CypD-deficient neurons were resistant to Aβ-induced p38 phosphorylation (**Fig. 5A–B**). It was noted that the baseline of phospho-p38 or total p38 in *Ppif* ^−/−^ neurons was comparable to that in nonTg neurons, suggesting no effect of CypD deficiency on p38 signal transduction under physiological condition. Based on the observations that CypD depletion significantly stabilized Aβ-induced intracellular calcium and ROS perturbations in neurons and elevated intracellular calcium and ROS are associated with axonal mitochondrial transport and dynamics defects, we then tested whether CypD deficiency inhibits A23187-induced p38 activation by stabilizing intracellular calcium levels and whether Aβ-induced p38 activation is suppressed by antioxidant. We first exposed nonTg and CypD deficient neurons to 5 µM A23187. Indeed, A23187 treatment significantly increased phospho-p38 compared to vehicle-treated nonTg neurons (Fig. S2). No significant elevation of p38 phosphorylation was detected in A23187-treated CypD deficient neurons (Fig. S2). Similarly, the co-incubation of Probucol with Aβ significantly suppressed Aβ-induced p38 activation in nonTg neurons (Fig. S3). Taken together, these data suggest the linkage of p38 activation with mPTP associated intracellular calcium and Aβ-induced ROS disturbances.

To determine if there is a direct link of p38 activation to mitochondrial transport, we assessed the effect of p38 inhibitor on axonal mitochondrial transport following Aβ treatment. Treatment with a specific p38 inhibitor (SB203580) resulted in a significantly higher percentage of movable mitochondria in Aβ-insulted neurons than in neurons without SB20358 treatment (**Fig. 5C**; 35.34±2.74% with SB203580 vs. 21.94±2.95% without SB203580). Similarly, SB203580 treatment protected against Aβ-induced alterations in both anterograde and retrograde mitochondria (**Fig. 5C**). As the result, SB203580 treatment attenuated Aβ-induced reduction in axonal mitochondrial density (**Fig. 5D**; 0.199±0.01/µm with SB203580 vs. 0.283±0.02/µm without SB203580). These results demonstrate that CypD depletion reduces Aβ-mediated activation of p38 contributing to the impairment of axonal mitochondrial transport.

### CypD Depletion Protects Against Aβ-induced Synaptic Damage

**Figure 6:**
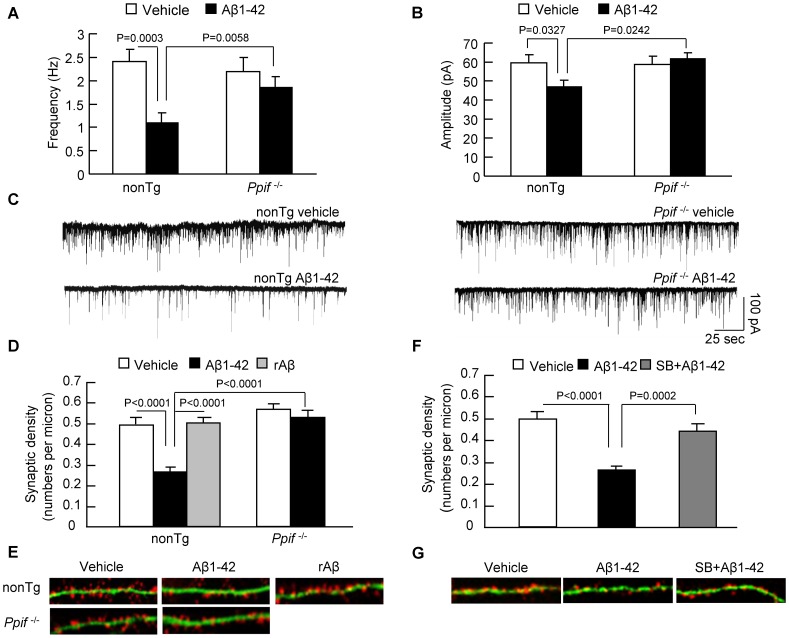
Effect of CypD on Aβ-induced synaptic damage. (**A–C**) Electrophysiological recording of mEPSCs for Aβ-treated nonTg and *Ppif* ^−/−^ neurons. CypD deficiency alleviated Aβ-induced decrease in mEPSCs frequency (**A**) and amplitude (**B**). Data were derived from 16–19 neurons for each group. (**C**) Representative traces of mEPSCs in the indicated group. Scale bar represents 100 pA in amplitude and 25 seconds in time. (**D–E**) Effect of CypD deficiency on synaptic density. The results were derived from 20–30 neurons of each group. Dendrites were visualized by the staining of MAP2 and synapses were recognized as synaptophysin-positive clusters overlapping with dendrites. (**E**) Representative images for double staining of synaptophysin and MAP-2 in the indicated groups. MAP2 is shown in green color and synaptophysin is labeled by red fluorescence. (**F–G**) Effect of Aβ-induced activation of p38 MAP kinase on synaptic density. (**F**) Administration of p38 inhibitor, SB203580 (1 µM, 24 hours) to cells ameliorated Aβ-induced synaptic loss. (**G**) Representative images showed the protected effects of synaptic density after SB203580 treatment.

To analyze the contribution of abnormal axonal mitochondrial transport and/or its directionality to synaptic dysfunction and loss of synapses in Aβ-rich environment, we measured synaptic activity by recording the spontaneous miniature excitatory post-synaptic currents (mEPSCs) and also quantified synaptic density. To determine the effect of CypD on synaptic activity, nonTg and *Ppif* ^−/−^neurons were treated with Aβ and then subjected to whole-cell patch-clamp recording of mEPSCs. The frequency of mEPSCs is largely associated with the probability of presynaptic release and the amplitude of mEPSCs at certain levels relies on the size of the vesicle-releasing pool in presynaptic regions [47]–[49]. Vehicle-treated nonTg and *Ppif* ^−/−^ neurons showed similar patterns of mEPSCs frequency and amplitude, suggesting no effect of CypD deficiency on the spontaneous nonaction potential-dependent activation of synapses under physiological condition. However, Aβ-insulted nonTg neurons showed a 54.6% decrease in mEPSCs frequency, compared to 16.4% reduction in *Ppif* ^−/−^ neurons (**Fig. 6A**). As a result, Aβ-superimposed *Ppif* ^−/−^ neurons significantly preserved mEPSCs frequency (**Fig. 6A, 6C**; 1.84±0.24 Hz in *Ppif* ^−/−^ neurons vs. 1.09±0.23 Hz in nonTg neurons). Similarly, the amplitude of mEPSC was significantly increased in Aβ-treated *Ppif* ^−/−^ neurons compared to Aβ-treated nonTg neurons (**Fig. 6B–C**; 61.88±3.05 pA in *Ppif* ^−/−^ vs. 47.03±3.28 pA in nonTg neurons).

To examine the protective effect of CypD depletion on Aβ-induced loss of synapses, we quantified synaptophysin-positive clusters attaching to dendrites in cultured hippocampal neurons derived from nonTg and CypD-deficient mice. Synapses were recognized as synaptophysin-positive clusters attaching to dendrites and dendrites were determined by MAP2 (microtubule-associated protein 2) staining. Aβ-treated nonTg neurons exhibited significantly decreased presynaptic density compared to vehicle-treated control (**Fig. 6D–E** **;** vehicle: 0.492±0.029/µm vs Aβ: 0.273±0.02/µm), whereas CypD depletion completely reversed the loss of presynaptic density (**Fig. 6D–E** **;** 0.527±0.026/µm). The rAβ did not affect synaptic density (**Fig. 6D–E**; 0.506±0.019/µm). There was no difference in presynaptic density between nonTg and *Ppif* ^−/−^ neurons in the vehicle-treated groups (**Fig. 6D–E**). To determine effect of p38 activation on loss of synapses, neurons were treated with specific p38 inhibitor (SB203580) for 30 min prior to Aβ. A shown in Fig. 6C–D, the addition of SB203580 to culture increased synaptic density (**Fig. 6F–G** **;** 0.442±0.033% with SB203580 vs. 0.273±0.020% without SB203580). Taken together, our results indicate that lack of CypD protects neuron from Aβ-insulted synaptic injury with involvement of CypD/Aβ-associated P38 MAPK signaling, which is associated with compromised mitochondrial transport in axon.

## Discussion

Abnormal axonal mitochondrial transport is a recently recognized mitochondrial pathology induced by Aβ [20]–[26], [50]. The precise mechanisms underlying impairments in axonal mitochondrial transport and the link of mitochondrial dysfunction to synaptic damage in AD are not well understood. In this study, we analyzed the effect of CypD on Aβ-mediated mitochondrial motility and distribution in hippocampal neurons using mice with genetic depletion of CypD. Our results show that CypD depletion protects against Aβ-induced alterations in axonal mitochondrial transport as shown by increased mitochondrial motility and distribution, and improved anterograde and retrograde movement. The possible mechanisms underlying the protective effect of lacking CypD are suppressed mPTP opening, reduced ROS production, and increased calcium buffering capacity in axonal mitochondria. Furthermore, we also demonstrated that CypD-mediated p38 activation contributes to Aβ-impaired axonal mitochondrial transport and synaptic injury. We focus our attention on the protective effect of CypD deficiency on axonal mitochondrial movement in view of the essential role of normal axonal mitochondrial trafficking in supporting synaptic plasticity. Our current study uncovers the role of CypD in Aβ-mediated alterations in axonal mitochondrial motility and dynamics contributing to synaptic degeneration in AD.

An increasing body of evidence suggests that oligomeric Aβ inhibits axonal mitochondrial transport and breaks the mitochondrial fusion/fission balance. Aβ-disrupted axonal mitochondrial trafficking is a mechanism underlying synaptic degeneration in AD [20], [23]–[26], [50]. In the present of study, we examined the effect of relatively low concentration of Aβ (200 nM) that did not alter cell viability on axonal mitochondrial transport to mimic low *in vivo* levels and chronic Aβ insults in AD brain. Similar to what have been reported [20], [23]–[26], [50], under our experimental condition, 200 nM oligomeric Aβ significantly reduced mitochondrial density and movement in axon by 30–40% (Fig. 1A–D and 2A) without significant changes in the cell viability. This suggests an early change in axonal mitochondrial trafficking is prior to neuronal death. A relatively low concentration of Aβ (200 nM) used in our study may account for the modest effects on mitochondrial movement without significant neurotoxicity. Indeed, a study has shown that the acute treatment of monomeric Aβ demonstrated significant inhibitory effect on neuronal mitochondrial movement [51], suggesting that both Aβ species (monomeric or oligomeric forms) are toxic to neuronal mitochondrial transport. In consideration of the significance of oligomeric Aβ-induced mitochondrial and synaptic dysfunction relevant to the AD pathogenesis [52] and our experimental condition (chronic treatment of low concentration of 200 nM Aβ for 24 hours) in which condition that monomeric Aβ is prone to form oligomers during incubation time [53], we used oligomeric Aβ for all our experiments. In addition, reversed Aβ peptide (rAβ) that has the same molecular weight and composition of amino acids with Aβ but without biological effects was used as a widely accepted control to verify the specific effects of Aβ [54], [55].

To elucidate the protective mechanisms of CypD depletion, we focused on the major consequences of mPTP formation on axonal mitochondrial motility and morphology: impaired mitochondrial calcium handling capacity and ROS generation. Aβ has been reported to increase intracellular Ca^2+^, which could have more targets than mitochondrial trafficking. In view of the role of CypD-dependent mPTP on maintaining intracellular Ca^2+^ homeostasis, significance of Aβ-impaired mitochondrial transport on synaptic degeneration, and unexplored role of CypD on mitochondrial transport, it is essential and logical to investigate the involvement of CypD on Aβ-induced abnormal axonal mitochondrial transport. CypD is a key component for the formation of mPTP contributing to maintaining calcium homeostasis. CypD deficiency inhibits opening of mPTP, subsequently, increases mitochondrial calcium buffering capacity in response to changes in intracellular calcium levels such as calcium overloading [12], [27], [29], [56]. Therefore, CypD-dependent mPTP is an important regulating mechanism of intracellular Ca^2+^ homeostasis.

We have presented data showing that blockade of CypD by genetic depletion of CypD or pharmacological CypD inhibitor significantly suppressed Aβ-induced elevation of the intracellular calcium in axon (Fig. 3A), which are consistent with our [12] and other [27] published studies. These results suggest that an inhibitory effect of CypD deficiency on Aβ-mediated changes in intracellular Ca^2+^ levels is important for maintaining normal mitochondrial transport. To test this hypothesis, we examined a direct effect of CypD deficiency on ionomycin (A23187)-induced Ca^2+^ overload, a strong inducer of Ca^2+^ elevation in intact cells, and alterations in axonal mitochondrial transport [27]. As expected, CypD-deficient neurons blocked A23187-induced elevation of intracellular Ca^2+^ (Fig. S4) and p38 activation (Fig. S2). This could be a mechanism of the protective effect of CypD deficiency on A23187-altered axonal mitochondrial trafficking (Fig. 3C–G). Compared to the effect of Aβ, A23187 treatment had a greater effect on mitochondrial transport (50% decline in A23187 treatment vs. 30–40% in Aβ-treated cells). A strong induction of calcium elevation in intact cells (high levels of Ca^2+^) by A23187 could be the explanation for a more dramatic effect of A23187 (50% in **Fig. 3C, E–F**) than Aβ treatment (30% in **Fig. 1A–B, C** **2, D1** and 40% in **Fig. 2A–C**) in which elevated levels of Ca^2+^ are expected to be lower than A23187-treated cells. A direct role of intracellular calcium in controlling axonal mitochondrial motility and dynamics is also supported by the recent study. For example, calcium-induced mitochondrial dissociation has been postulated as a potential mechanism for modulation of mitochondrial docking under physiological conditions [57]. Increased calcium levels are reported to decrease mitochondrial movement/transport by interrupting Miro and kinesin complexes [57], [58]. At pathological states with significant and sustained calcium elevation achieved by the activation of ***N***-Methyl-D-aspartate (NMDA) receptors [37] or the application of A23187 [59], mitochondrial morphology and movement are substantially disrupted, suggesting the impact of pathological intra-calcium perturbations. In Aβ-rich environment where calcium levels are abnormally high [60]–[63], increased mitochondrial detachments occur in Aβ-treated axons (represented by an increased percentage of stationary mitochondria). Thus, axonal mitochondria are crucial to the calcium buffering process. Maintenance of axonal calcium homeostasis by CypD depletion is an underlying mechanism for controlling axonal mitochondrial calcium in the face of Aβ insults. Aβ-mediated elevation of calcium is a potential mechanism at the nexus of Aβ toxicity and alterations in mitochondrial motility. The dramatic protection of lacking CypD against A23187-disturbed calcium balance as well as mitochondrial motility and dynamics changes provides substantial evidence that the blockade of CypD-mediated mPTP counteracts calcium-instigated axonal mitochondrial alterations in trafficking and morphology.

Another major consequence of CypD-mediated mPTP formation is increased ROS production/accumulation leading to release of ROS from mitochondrial to cytosol. As Ca^2+^ metabolism and oxidative stress are intertwined, especially in mitochondrial processes, these organelles can become severely dysfunctional during the permeability transition in combination with effects of oxidative stress and dysregulation of cytosolic free Ca^2+^. Indeed, in the present study, we report reduced mitochondrial calcium buffering capacity, increased membrane permeability transition, and accumulation of ROS in axons in the presence of Aβ. ROS has been implicated in disruption of mitochondrial movement. For example, zinc-induced ROS generation is associated with phosphatidylinositol (PI) 3-kinase activation, which in turn disrupts mitochondrial transport in neurons [64]. However, to our knowledge, the contribution of axonal ROS dysregulation to Aβ-induced defects in mitochondrial transport has not yet been documented. We showed here that the addition of probucol, an antioxidant drug to suppress ROS generation, or genetic deletion of CypD to blunt oxidative stress and to enhance mitochondrial calcium buffer capability significantly rescues mitochondrial movement against Aβ toxicity, indicating the significance of oxidative stress on Aβ-altered axonal mitochondrial trafficking. These data support that Aβ-induced intra-axonal ROS has deleterious effects on transport.

Activation of P38 mitogen-activated protein kinase (MAPK) is associated with increased intracellular calcium, ROS production/accumulation, Aβ stimulation, and mitochondrial stress [43], [65]–[71]. We demonstrated that levels of P38 phosphorylation were significantly increased in Aβ-treated neurons. Antioxidant Probucol blocked Aβ-induced p38 activation, indicating a role of Aβ-induced oxidative stress in disruption of signal transduction such as p38 MAP kinase contributing to abnormal axonal mitochondrial transport. Notably, Aβ-induced p38 phosphorylation was blunted in neurons lacking CypD. The addition of a specific p38 inhibitor (SB203580) resulted in pronounced preservation of mitochondrial motility and morphology even in the face of Aβ insults, indicating the involvement of CypD/Aβ-associated p38 MAPK signaling in disruption of axonal mitochondrial trafficking. The application of p38 inhibitor did not interfere with Aβ-induced calcium elevation (data not shown). These results suggest that p38 is a downstream target of Aβ. Thus, we propose that CypD-dependent impaired calcium homeostasis and ROS production/accumulation in axons are responsible for p38 MAPK activation, which leads to further mitochondrial injury including abnormal axonal mitochondrial transport and loss of synapse. The detailed mechanisms of P38 activation in injuring axonal mitochondrial transport need further investigation. For example, P38 activation is connected with changes in mitochondrial movement via phosphorylation of kinesin [46] and dynein [72], which dissociates mitochondria from the motor proteins. In addition to p38, perturbations of several other signaling cascades including PKA [51] and GSK-3β [23] are also reported to be involved in Aβ-induced disruption in mitochondrial transport. Given the tight interaction of these signaling cascades [73], they may work together in keeping axonal mitochondrial movement in normal fashion while their independent effects on mitochondrial trafficking remain unclear. In view of potential involvement of motor proteins in mitochondrial movement, we will further examine whether mPTP-associated axonal mitochondrial transport changes are related to changes of motor proteins such as hyperphosphorylation of dynein and kinesin and altered Miro activity state in near future. Nevertheless, our study suggests that CypD-involved activation of p38 signaling plays a role, at least in part, in Aβ-insulted abnormal mitochondrial transport in axon.

Axonal mitochondria are dynamic organelles and their trafficking and docking are critical for synaptic plasticity and function. Synaptic loss and deactivation are biological basis of AD. Increasing evidence emphasizes the importance of mitochondria for the maintenance of synaptic function. Defects in dendritic mitochondria lead to dendritic degeneration [74] and injured mitochondria in the presynapse region are associated with compromised presynaptic function [75]. Mitochondrial transport maintains functional mitochondria around synapses [76] Previously, we and other groups showed that Aβ insults results in impaired mitochondrial distribution and trafficking in axons [20], [23]–[26], while in the present study, we demonstrate the protective effects of CypD depletion on Aβ-mediated deficits in axonal mitochondrial transport and synaptic injury including synaptic activity and loss of synapses. Notably, blockade of p38 activation significantly rescue synaptic loss insulted by Aβ (Fig. 6F–G), supporting a connection of CypD/Aβ-involved signal transduction (p38) with mitochondrial and synaptic degeneration.

**Figure 7:**
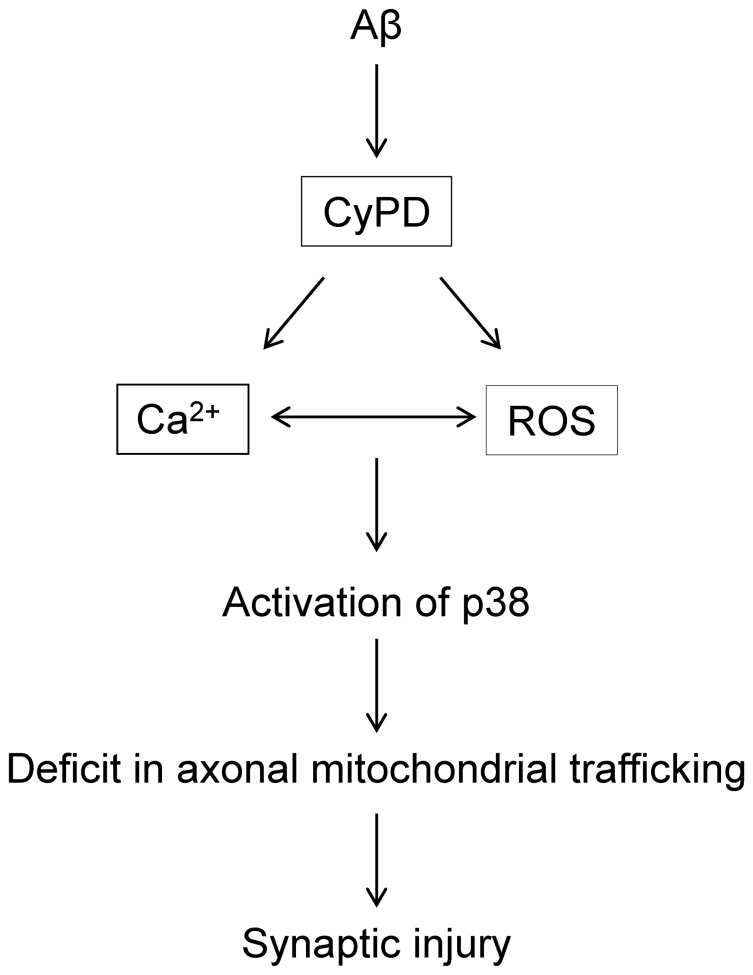
Working hypothesis. Aβ-Cyclphilin D mediates impairments in axonal mitochondrial transport. In the present of Aβ, there is an increase in the opening of CypD-mediated mitochondrial permeability transition pore (mPTP), leading to disruption of Ca^2+^ balance and increase in reactive oxygen species (ROS) production/accumulation. Consequently, elevation of Ca2+ and oxidative activates downstream signal pathway p38 MAP Kinase contributing to mitochondrial dysfunction, deficits in axonal mitochondrial trafficking, eventually, synaptic damage.

In summary, our data offer new insights into the mechanism of mitochondrial perturbation in the pathogenesis of AD, specifically the role of CypD in axonal mitochondrial transport. Aβ-CypD interaction promotes opening of mitochondrial permeability transition pore, consequently, disrupts calcium balance and enhances production/accumulation of ROS, thereby further activating P38 MAPK signal transduction pathway. All these events disrupt mitochondrial trafficking and dynamics, ultimately causing synaptic damage (**Fig. 7**). We have clearly demonstrated that CypD depletion protects axonal mitochondrial transport from Aβ insults along with suppressing Aβ-induced elevation of calcium and accumulation of oxidative stress. Importantly, CypD depletion also suppressed Aβ-induced activation of p38/MAPK and this inhibition rescued axonal mitochondrial movement and presynaptic density. Thus, our results provide evidence that CypD/Aβ-mediated mitochondrial dysfunction is correlated with disruption of axonal mitochondrial transport and synaptic injury. These findings significantly enhance our understanding of the pathological role of CypD in axonal pathology in AD.

## Supporting Information

Figure S1 Cultured hippocampal neurons were transfected with pDsRedmito and observed under microscope. The figure showed the image of a transfected neuron. Middle part of the axon (in the frame) was selected for the experiment to detect mitochondrial movement.
(TIF)

Figure S2 CypD depletion suppresses A23187-induced p38 phosphorylation. NonTg and CypD deficient hippocampal neurons were exposed to 5 µM A23187 for 15 and 30 min, respectively. Cell lysates were subjected to immunoblots for phospho- and total-p38. The treatment of A23187 on nonTg neurons significantly increased p38 phosphorylation level as compared to the vehicle-treated neurons (vehicle: 1±0.021 vs. A2318715 min: 1.89±0.047; vehicle vs. A23187 30 min: 2.06±0.18). CypD depletion significantly suppressed A23187-induced elevation of phospho-p38. Data were derived from 4 independent experiments.
(TIF)

Figure S3 Addition of Probucol attenuates Aβ-induced p38 phosphorylation in nonTg neurons. nonTg neurons were treated with Aβ co-incubated in the presence or absence of Probucol (5 µM, 24 hours). Aβ treatment resulted in a significant elevation of p38 phosphorylation level as compared to vehicle treatment (vehicle: 1±0.077 vs. Aβ: 3.06±0.27), while Aβ-induced p38 phosphorylation was inhibited by the addition of Probucol (Aβ: 3.06±0.27 vs. Aβ+Probucol: 1.15±0.46). Data were derived from 3 independent experiments.
(TIF)

Figure S4 Effect of CypD depletion on A23187-induced intra-axonal calcium elevation. NonTg and *Ppif* ^−/−^ hippocampal neurons were exposed to A23187 (5 µM for 30 min) and subjected to recording of intra-axonal calcium before and after A23187 treatment. A23187 treatment resulted in increased axonal calcium level in nonTg neurons. *Ppif* ^−/−^ neurons abolished A23187-induced calcium elevation.
(TIF)
