# Supplementary figures and images for: Cyclophilin D Deficiency Rescues Axonal Mitochondrial Transport in Alzheimer’s Neurons
Source: PLoS One. 2013 Jan 31;8(1):e54914. doi: 10.1371/journal.pone.0054914 (PMC3561411; doi:10.1371/journal.pone.0054914)

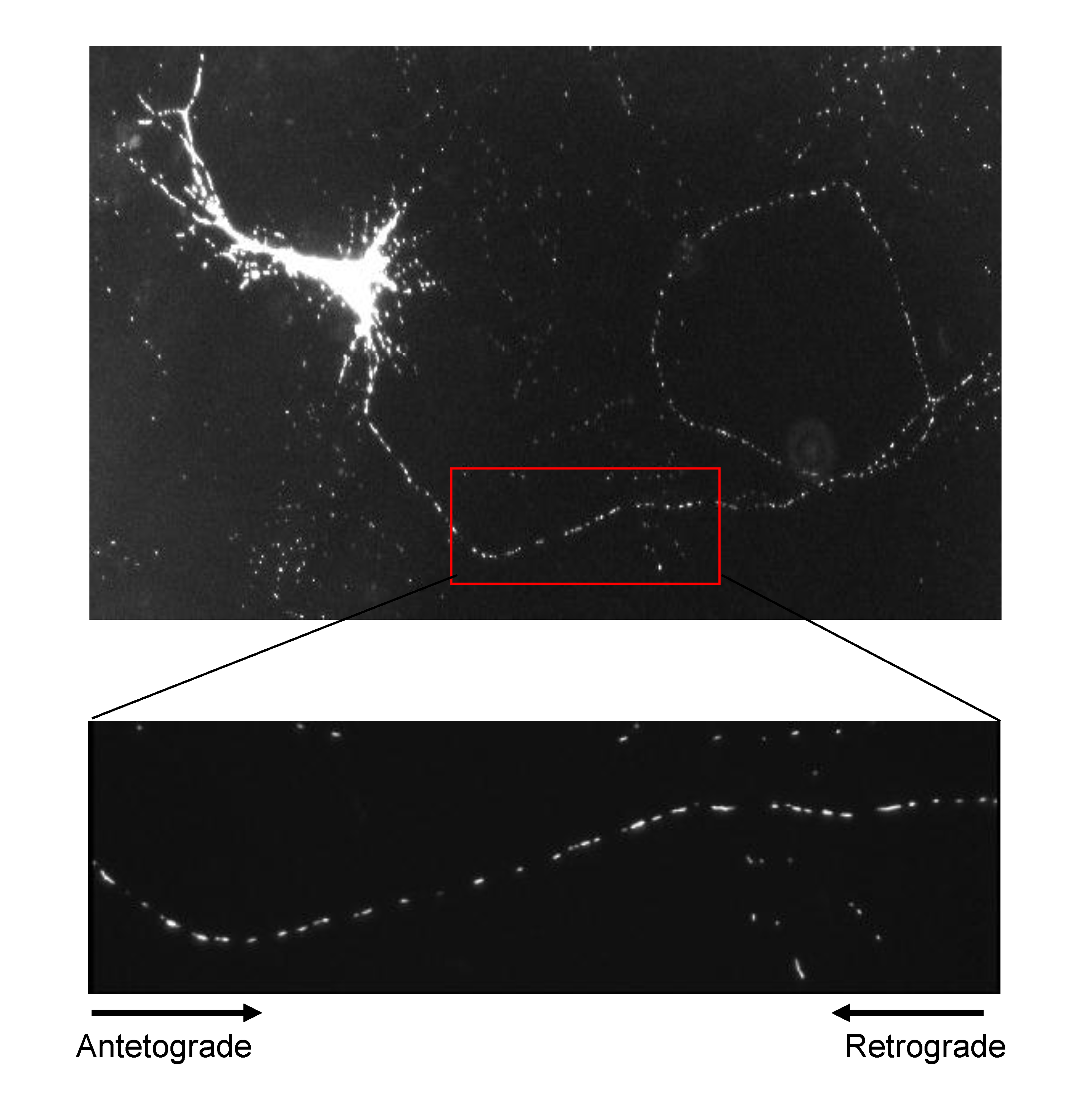

Supplement: Figure S1 — Cultured hippocampal neurons were transfected with pDsRedmito and observed under microscope. The figure showed the image of a transfected neuron. Middle part of the axon (in the frame) was selected for the experiment to detect mitochondrial movement. (TIF) [file pone.0054914.s001.tif]

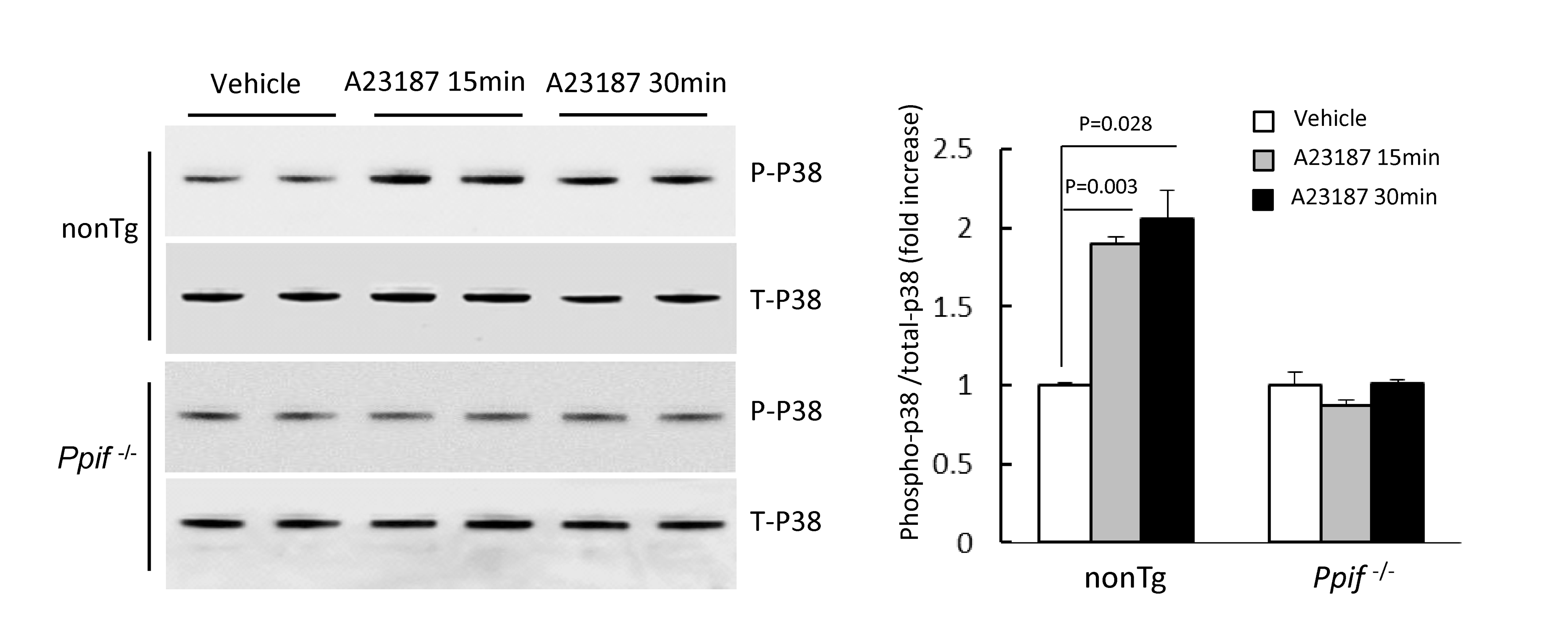

Supplement: Figure S2 — CypD depletion suppresses A23187-induced p38 phosphorylation. NonTg and CypD deficient hippocampal neurons were exposed to 5 µM A23187 for 15 and 30 min, respectively. Cell lysates were subjected to immunoblots for phospho- and total-p38. The treatment of A23187 on nonTg neurons significantly increased p38 phosphorylation level as compared to the vehicle-treated neurons (vehicle: 1±0.021 vs. A2318715 min: 1.89±0.047; vehicle vs. A23187 30 min: 2.06±0.18). CypD depletion significantly suppressed A23187-induced elevation of phospho-p38. Data were derived from 4 independent experiments. (TIF) [file pone.0054914.s002.tif]

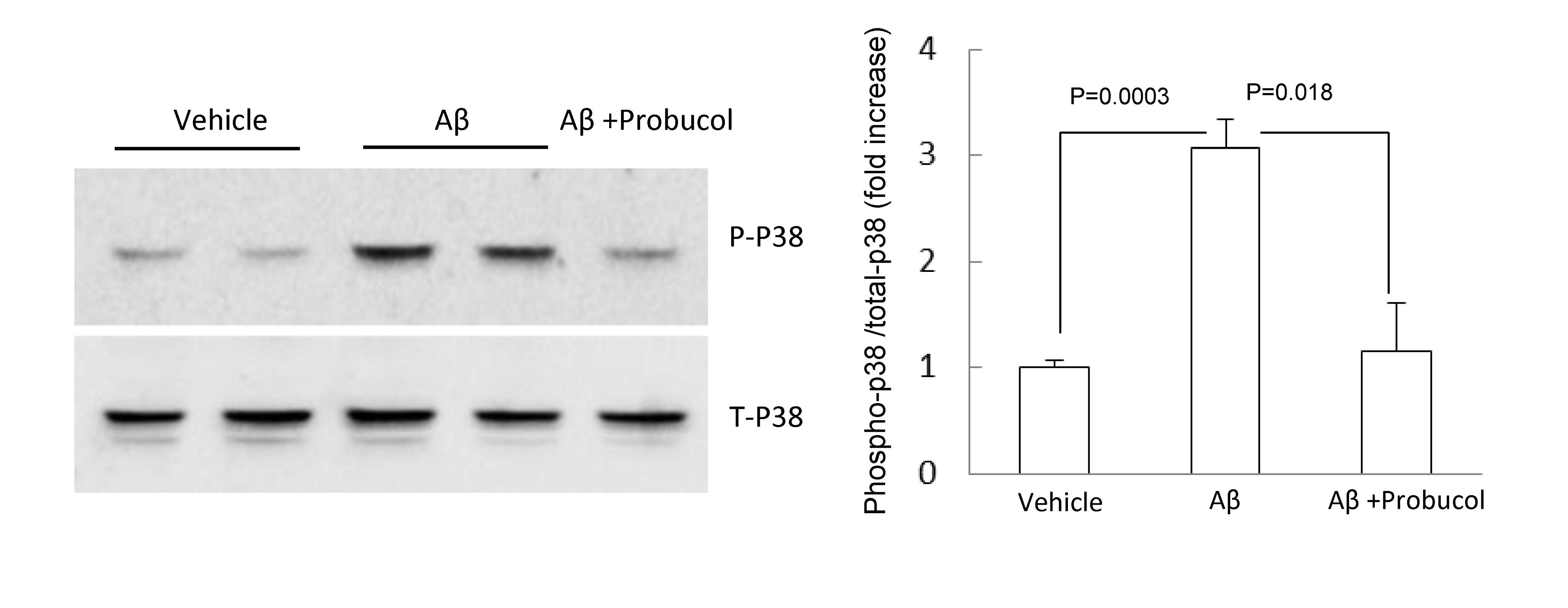

Supplement: Figure S3 — Addition of Probucol attenuates Aβ-induced p38 phosphorylation in nonTg neurons. nonTg neurons were treated with Aβ co-incubated in the presence or absence of Probucol (5 µM, 24 hours). Aβ treatment resulted in a significant elevation of p38 phosphorylation level as compared to vehicle treatment (vehicle: 1±0.077 vs. Aβ: 3.06±0.27), while Aβ-induced p38 phosphorylation was inhibited by the addition of Probucol (Aβ: 3.06±0.27 vs. Aβ+Probucol: 1.15±0.46). Data were derived from 3 independent experiments. (TIF) [file pone.0054914.s003.tif]

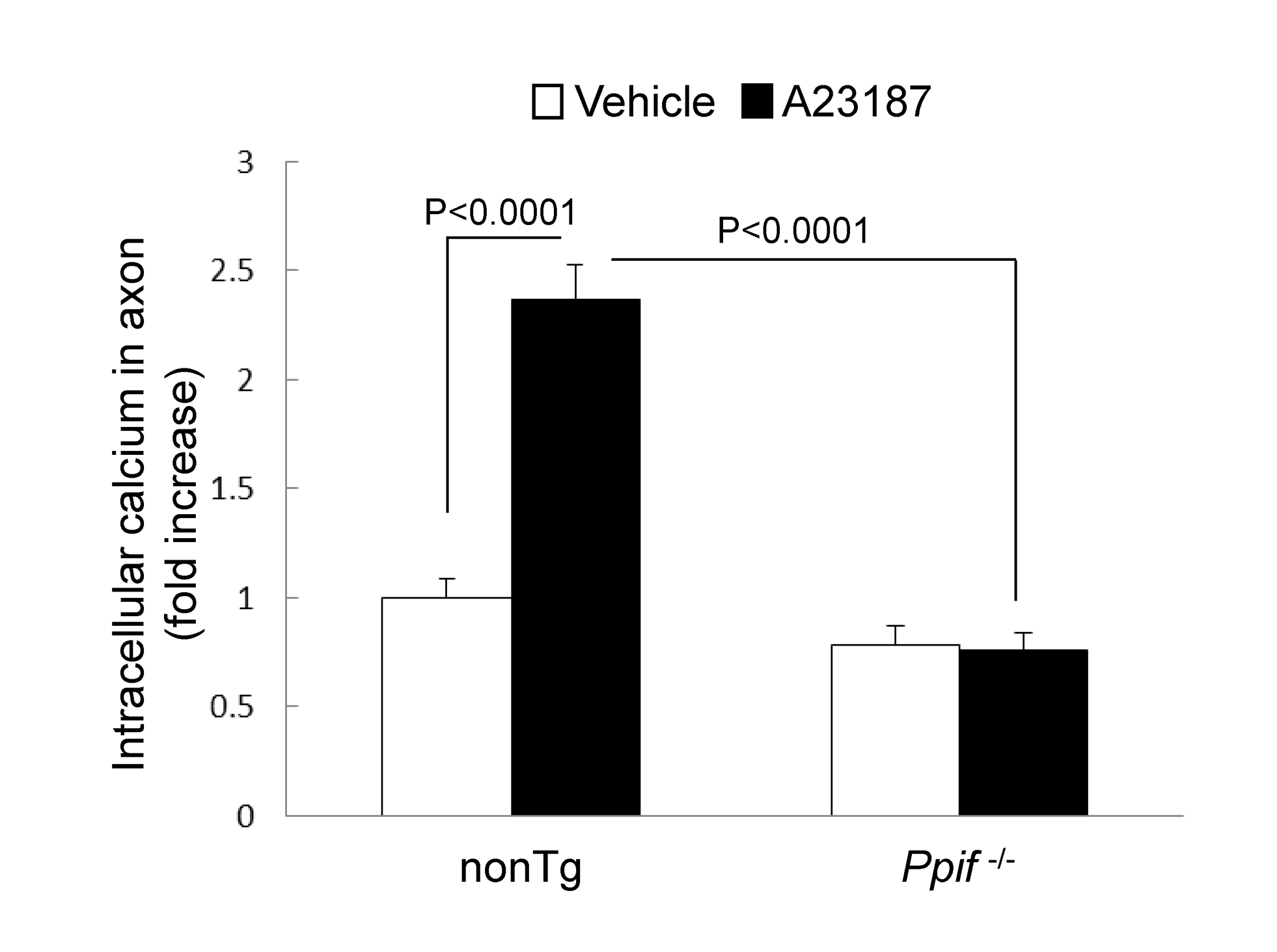

Supplement: Figure S4 — Effect of CypD depletion on A23187-induced intra-axonal calcium elevation. NonTg and Ppif −/− hippocampal neurons were exposed to A23187 (5 µM for 30 min) and subjected to recording of intra-axonal calcium before and after A23187 treatment. A23187 treatment resulted in increased axonal calcium level in nonTg neurons. Ppif −/− neurons abolished A23187-induced calcium elevation. (TIF) [file pone.0054914.s004.tif]
